# Heloise

**Published:** 1887-09

**Authors:** 


### HELOISE.

Have a care little maid, will you never give over
  Bewitching our hearts with those merry blue eyes,
That laugh all the same when they've captured a lover,
  And care not a fig for his pleading and sighs.
      Oh beware, have a care !
There's a little winged archer that ranges the skies.

A little winged archer with quiver and bow,
  Whose sight is so keen and whose aim is so true
Every shaft to the centre is certain to go,
  So prepare little maid with eyes merry and blue,
      Oh beware, have a care !
That little winged archer is looking for you.

De Travers.

